# Clinical Evaluation of a Microwave-Based Device for Detection of Traumatic Intracranial Hemorrhage

**DOI:** 10.1089/neu.2016.4869

**Published:** 2017-07-01

**Authors:** Johan Ljungqvist, Stefan Candefjord, Mikael Persson, Lars Jönsson, Thomas Skoglund, Mikael Elam

**Affiliations:** ^1^ Department of Neurosurgery, Sahlgrenska University Hospital, Gothenburg, Sweden.; ^2^ Institute of Neuroscience and Physiology, Department of Clinical Neuroscience, The Sahlgrenska Academy at the University of Gothenburg, Gothenburg, Sweden.; ^3^ Department of Signals and Systems, Chalmers University of Technology, Gothenburg, Sweden.; ^4^ MedTech West, Sahlgrenska University Hospital, Gothenburg, Sweden.; ^5^ SAFER Vehicle and Traffic Safety Centre at Chalmers, Gothenburg, Sweden.; ^6^ Department of Neuroradiology, Sahlgrenska University Hospital, Gothenburg, Sweden.; ^7^ Department of Clinical Neurophysiology, Sahlgrenska University Hospital, Gothenburg, Sweden.

**Keywords:** chronic subdural hematoma, intracranial hemorrhage detection, microwave technology, traumatic brain injury, triage

## Abstract

Traumatic brain injury (TBI) is the leading cause of death and disability among young persons. A key to improve outcome for patients with TBI is to reduce the time from injury to definitive care by achieving high triage accuracy. Microwave technology (MWT) allows for a portable device to be used in the pre-hospital setting for detection of intracranial hematomas at the scene of injury, thereby enhancing early triage and allowing for more adequate early care. MWT has previously been evaluated for medical applications including the ability to differentiate between hemorrhagic and ischemic stroke. The purpose of this study was to test whether MWT in conjunction with a diagnostic mathematical algorithm could be used as a medical screening tool to differentiate patients with traumatic intracranial hematomas, chronic subdural hematomas (cSDH), from a healthy control (HC) group. Twenty patients with cSDH and 20 HC were measured with a MWT device. The accuracy of the diagnostic algorithm was assessed using a leave-one-out analysis. At 100% sensitivity, the specificity was 75%—i.e., all hematomas were detected at the cost of 25% false positives (patients who would be overtriaged). Considering the need for methods to identify patients with intracranial hematomas in the pre-hospital setting, MWT shows promise as a tool to improve triage accuracy. Further studies are under way to evaluate MWT in patients with other intracranial hemorrhages.

## Introduction

Early detection and evacuation of space-occupying intracranial hematomas is one of the cornerstones in the treatment of patients with traumatic brain injury (TBI). Seelig and associates^1^ showed that in patients with traumatic acute subdural hematomas, the delay from injury to operation was the factor of greatest therapeutic importance. Diagnosis and removal of the hematoma within four hours of injury considerably reduced death—i.e., from 90% to 30% mortality rate (*n* = 82, *p* < 0.0001). Any further delay in hematoma evacuation severely increased death and worsened functional outcome in the patients who survived.^1^

A key to improve outcome for patients with TBI is to reduce the time to definitive care by achieving high triage accuracy. Unfortunately, this is not always attained in contemporary emergency care. Xiang and colleagues^2^ showed that more than one third of patients with major trauma (Injury Severity Score [ISS] ≥16) in the United States were undertriaged, with TBI constituting >40% of undertriaged diagnoses, and intracranial hemorrhage being top-ranked. Haas and coworkers^3^ showed that death was almost 25% higher for patients transported to a non-trauma center compared with direct transport to a trauma center (*n* = 11,398, odds ratio 95%, confidence interval 1.10–1.40). Transporting all patients with suspected TBI to a center with neurosurgical expertise, however, results in substantial overtriage, which addresses the need for reliable TBI diagnosis at the scene of injury.^4^

Computed tomography (CT) is the gold standard for detection of intracranial hematomas. In most cases, however, the patient must wait until arrival at a hospital with access to radiology facilities before a CT scan can be performed. A compact system that can detect intracranial hemorrhage in a pre-hospital setting—e.g., in road and air ambulances—could improve triage accuracy and reduce the time between injury and operation, resulting in reduced mortality rates and improved functional outcomes. Several different technologies have been investigated with the aim to develop such a system.^5–8^

Microwave technology (MWT) for biomedical applications has been explored for more than three decades.^9^ Driving forces include the potential to realize portable devices at low cost to convey diagnostic information in a fast, non-invasive, and safe manner. This can be accomplished by employing non-ionizing microwave radiation with low power in conjunction with effective mathematical algorithms for signal processing. Because of the scattering nature of microwaves propagating in inhomogeneous media such as tissue, computationally demanding algorithms are required to process the data. Previous lack of sufficient computational power is one reason for the delay in introducing MTW in clinical settings.

During the last decade, clinically relevant results have been realized, mainly for breast cancer.^10–12^ MWT can detect lesions such as cancer and internal bleedings because of the dielectric contrast between tissue types.^13,14^ For detection of intracranial hemorrhage, the contrast between blood and brain matter is utilized.^15^ In neurosurgical care, MWT could be used to monitor patients with conservatively managed hematomas, to monitor patients post-operatively, and to monitor patients with trauma in neurointensive care who are at risk of progressing contusions or extracerebral hematomas.

Recently, Persson and associates^15^ showed that MWT can differentiate hemorrhagic and ischemic stroke. They used two MWT prototype systems to measure 20 and 25 stroke patients, respectively, in the first two proof-of-principle clinical studies. A machine-learning approach was used to differentiate the two patient groups—i.e., a classifier was derived from supervised (patient diagnosis is known) training on the patient measurements and evaluated using a so-called leave-one-out (LOO) analysis.

Encouraging results were presented, achieving areas under the receiver operating characteristic (ROC) curve (AUC) of 0.88 and 0.85 for the first and second clinical trial, respectively. Sensitivity and specificity depend on the classifier threshold (decision offset). For example, at 90% sensitivity for detection of hemorrhagic stroke, the specificity was 65% for the second clinical study.^15^ The ability of a device to differentiate hemorrhagic and ischemic stroke in the pre-hospital setting could allow for rapid thrombolytic therapy in patients with ischemia and more adequate care for patients with intracerebral hematomas.

The purpose of the current clinical investigation was to test whether MWT, using the first portable device enabling pre-hospital measurements, could be used as a medical screening tool to differentiate patients with a traumatic intracranial hematoma from a healthy control (HC) group. Because this is the first study using MWT to examine patients with trauma, we chose to include patients with chronic subdural hematomas (cSDH), which is an intracranial hemorrhage usually not necessitating immediate management. A cSDH is a collection of blood and blood breakdown products between the surface of the brain and the dura. It is often caused by a minor head injury, expands slowly, and symptoms can take weeks to appear.^16^ Large or symptomatic hematomas are usually considered indications for surgical evacuation.^16^ By studying patients with cSDH, we aimed to test the viability of MWT for hematoma detection, in terms of sensitivity and specificity, compared to the CT scan as the gold standard.

## Patients and Methods

### Study subjects

This study was approved by the Regional Ethical Review Board at the University of Gothenburg, Sweden, and reviewed by The Medical Products Agency - Sweden. It was conducted according to Good Clinical Practice, the revised Declaration of Helsinki, and the ISO 14155:2011 standard. The study was registered at ClinicalTrials.gov (Identifier: NCT02282228) before the recruitment of the first study participant.

All patients were referred to Sahlgrenska University Hospital for operation of cSDH, during the period September 2015 to January 2016. The HC group was recruited in the same period as the patients and matched to the patient cohort for age and sex. Written informed consent was obtained from all participants before any study-related procedure was initiated. Safety follow-up for participants was performed >12 h after the diagnostic procedure. The inclusion and exclusion criteria are shown in Table 1.

**Table 1.** Inclusion and Exclusion Criteria for Patients and Controls

*Inclusion criteria patients*	*Inclusion criteria controls*	*Exclusion criteria patients and controls*
Admitted for surgery of cSDH	Healthy—i.e., no significant medical history	Pregnant or breastfeeding women
CT brain scan within 96 h	≥18 years of age	Shunt or other foreign object implanted in the brain
≥18 years of age	Signed a written informed consent	Participation in any other clinical study that could interfere with the result in the ongoing study
Be able to have a normal conversation and understand the information about the study, corresponding to Glasgow Coma Scale (Verbal Response) of 5		
Signed a written informed consent		

cSDH, chronic subdural hematoma; CT, computed tomography.

### Measurements

Eligible patients were included in the study on arrival at the neurosurgical unit. They were measured with the microwave device (Strokefinder MD100, Medfield Diagnostics AB, Gothenburg, Sweden) before operation of cSDH. Measurements were performed with the subjects lying in a hospital bed. A thin disposable plastic protective cover (evercare^®^ Banded Bag - REF 1855 - Ø 140 cm, OneMed Group Oy, Helsinki, Finland) was mounted around the device for hygienic reasons before the subject's head was positioned in the device. The subjects were required to lie still for the duration of the measurement (approximately 45 sec). No significant movements were visually observed during any subject measurements. Two investigators performed all measurements.

**FIG. 1.**
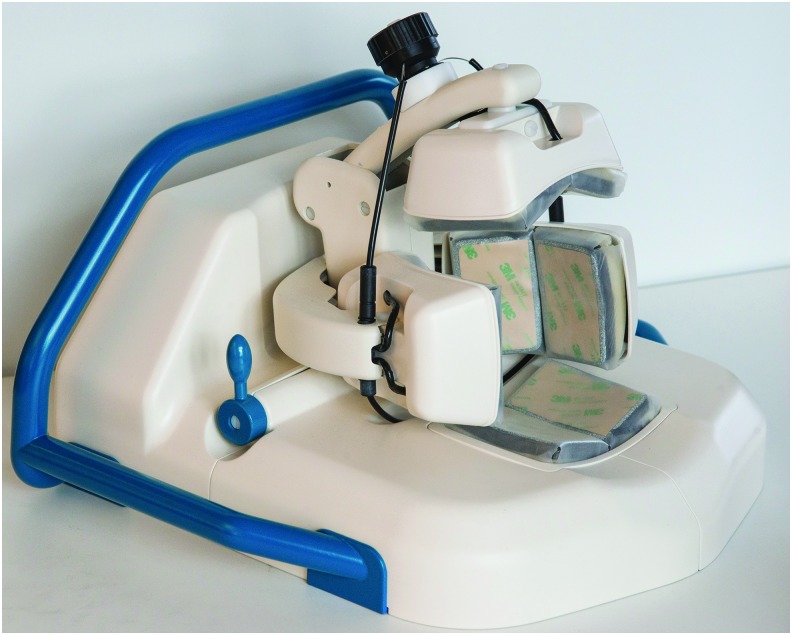
The Strokefinder MD100 device (Medfield Diagnostics AB, Gothenburg, Sweden). Reprinted with permission.

The Strokefinder MD100 is designed like a headrest for placement on a hospital bed or ambulance stretcher (Fig. 1). The device has eight antennas mounted in four pairs, where the pair in contact with the back of the patient's head is mounted at the bottom, and the other three pairs are mounted on separate arms. When the patient's head has been positioned in the device, the fastening mechanism is tightened until sufficient antenna contact with the head is achieved, without causing discomfort to the subject. The investigators followed a standardized procedure to position the patient's head, and carefully inspected that the head was placed symmetrically in the device. The measurements were started from a tablet computer, which was connected wirelessly to the main unit. The proprietary software included a test verifying that all antennas achieved good contact with the subject's head. If the test showed that one or more antennas did not achieve good contact, the subject's head was repositioned and the antenna positions and contact pressure controlled until the test was approved. The device automatically performed three consecutive measurements during the diagnostic procedure.

### CT-scan assessment

The patients' CT-scans were reviewed. Volume and attenuation of the hematomas and midline shift were measured. The volume of the hematomas was estimated by delineating the hematomas manually using the ITK-SNAP software (Version 3.4.0, University of Pennsylvania), and the software then automatically calculated the volume. A neuroradiologist (author LJ) then measured the attenuation of the hematomas and confirmed the accuracy of the volume and midline shift measurements.

### Microwave data pre-processing and analysis

**FIG. 2.**
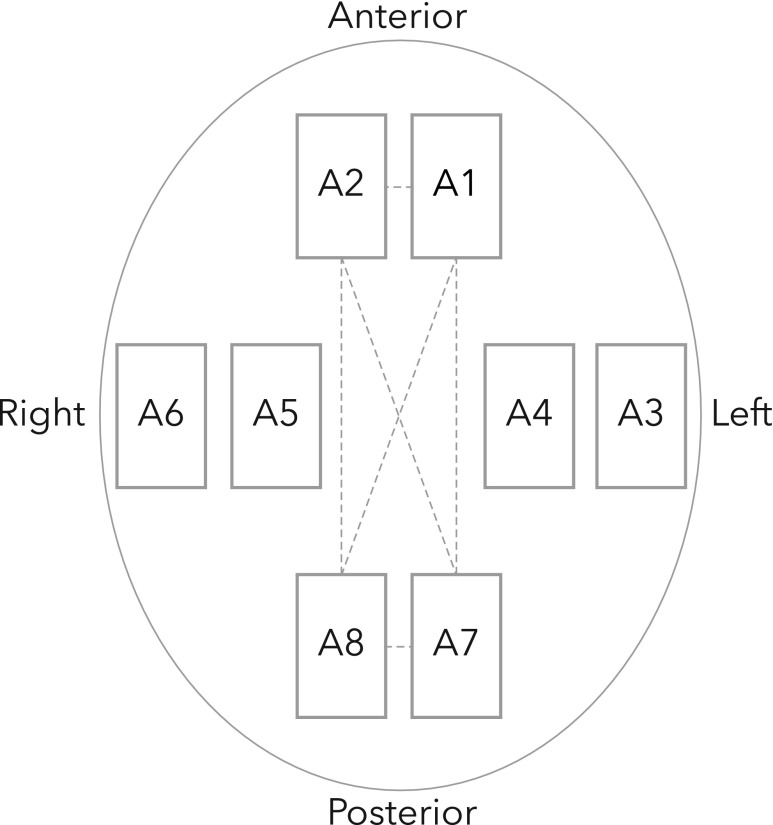
The reflection and transmission coefficients for antennas 1 and 2 (at subject's forehead) and antennas 7 and 8 (at back of subject's head) were removed, except for channels between the lateral antennas (3 to 6) and forehead/back of head antennas. The dashed lines indicate the channels that were removed.

The raw microwave data consisted of all antenna combinations (channels), including both reflection channels (same antenna sending and receiving) and transmission channels (one antenna sending, the other antennas receiving). The full frequency range was approximately 0.1–1.95 GHz. The raw data were pre-processed—i.e., prepared and transformed into a format suitable for the diagnostic algorithm, by excluding channels with high attenuation (i.e., low signal-to-noise ratio, because of a relatively long direct path through the brain) and channels that measured more peripheral regions relative to the commonly lateral location of cSDH. Figure 2 illustrates the excluded channels. For each measurement, all selected frequencies of the retained channels were combined into one complex vector. The three measurements on each subject were averaged into one mean measurement. No further pre-processing of the raw data was performed.

The classifier developed by Persson and coworkers^15^ was used on the pre-processed data. A LOO cross-validation procedure was used—i.e., the subject measurement to be classified was not included in the training data—to avoid overfitting. The classifier performance depends on which so-called subspace dimensions are retained.^15^ A repeated LOO procedure was used to identify the subspace dimensions and frequency interval that yielded optimal classifier performance.

The diagnostic performance was evaluated using the ROC and the AUC. An AUC of ∼0.5 represents a useless diagnostic test, equal to relying on chance. An AUC of 1.0 represents a perfect test that classifies all subjects correctly. The specificity at 100% sensitivity was derived from the ROC. A scatter plot was used to show the classifier decision value for all subjects.^15^ The frequency was swept from 0.1–1.95 GHz with 50 MHz step-size and a minimum frequency interval of 400 MHz. The highest AUC on average for all frequency intervals was given by keeping only the first subspace dimension, and this classifier setting was therefore used throughout all tests in this study. The frequency interval 0.75–1.95 GHz yielded the highest specificity at 100% sensitivity and was therefore used throughout all tests in this study.

To test the robustness of the classification with the selected pre-processing and classification parameter settings, 100 repeated LOO procedures were performed on data sets where a few random subjects were removed each time. We chose to remove two random subjects from the full data set for each iteration. The mean and standard deviation of the AUC were calculated from the 100 iterations. If the mean AUC is close to the AUC for the full data set and the standard deviation is relatively small, this indicates that the selected parameter settings for the classification are robust—i.e., the result does not fluctuate substantially depending on the exact configuration of the data set.

To validate that the classifier did not become overfitted (model being fitted to irrelevant information/noise) to the data, 100 repeated LOO procedures were performed on data sets where the subjects were randomly assigned to one of the two classes—i.e., each subject was assigned to either the cSDH group or the HC group regardless of the true diagnosis. For such random data sets, it is expected that the groups cannot be differentiated—i.e., an AUC ∼0.5 is expected. It is a useful check for bias of the mathematical model, which would cause deviations from the expected performance.^17^ The mean and standard deviation of the AUC were calculated from the 100 iterations.

## Results

**FIG. 3.**
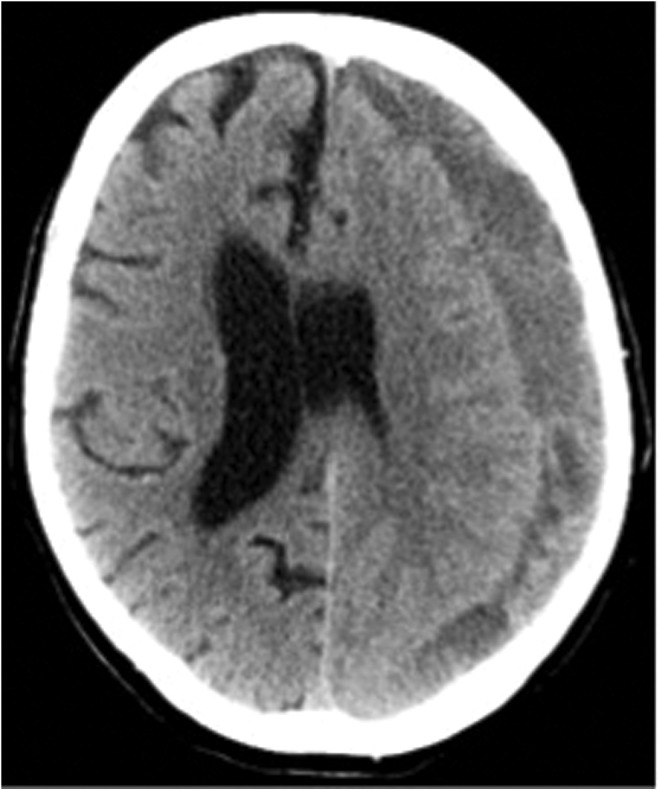
A typical appearance of a cSDH on the left side (patient P3) on a computed tomography scan, with its characteristic crescent-shaped form spanning over the coronal and lambdoid sutural margins. The hematoma is in the hypodense phase. Notice also the significant midline shift.

Twenty patients with cSDH and 20 HC subjects were included in the study. Table 2 shows age, sex, main symptoms, hematoma volume, attenuation, and midline shift for the patients. Eight female and 12 male patients were included, and the mean age for the patients was 75.6 years (range 54–90 years). The HC group consisted of nine females and 11 males with a mean age of 71.8 years (range 61–82 years). Figure 3 shows a representative example of a CT scan of a patient with cSDH.

**Table 2.** Characteristics of the Patients^*^

				*Hematoma volume*			
*Patient*	*Age (years)*	*Sex (male/female)*	*Main symptoms*	*Right (mL)*	*Left (mL)*	*Attenuation (HU)*	*Midline shift (mm)*
P1	63	M	Hemiparesis left side. Headache.	104	–	26	7
P2	90	F	Hemiparesis left side.	116	18	22–49	7
P3	81	M	Headache. Discrete dysphasia.	–	174	26–49	4
P4	67	M	Headache. Disturbed balance.	163	–	32–58	14
P5	87	M	Discrete hemiparesis right side.	–	67	24–54	1
P6	75	F	Discrete hemiparesis right side.	15	50	17–43	4
P7	86	M	Hemiparesis left side.	143	–	30	6
P8	67	M	Headache. Disturbed balance.	20	87	35–55	6
P9	81	M	Hemiparesis left side.	75	–	23–59	4
P10	76	F	Disturbed balance.	108	102	31–41	2
P11	65	M	Paresis right leg. Headache.	–	104	25–29	8
P12	67	M	Hemiparesis left side. Headache.	94	–	37–51	7
P13	88	F	Headache. Discrete dysphasia.	–	43	28–41	6
P14	63	M	Discrete dysphasia. Poor motor skills right hand.	–	114	50–61	8
P15	54	F	Headache.	27	–	30–39	4
P16	76	F	Headache.	39	22	22–54	0
P17	67	F	Discrete paresis left arm. Headache.	112	–	18	12
P18	86	M	Hemiparesis right side.	–	144	32–57	5
P19	88	F	Hemiparesis right side.	–	118	23–53	7
P20	85	M	Hemiparesis left side.	105	67	24–50	3

^*^ Age, sex, main symptoms, hematoma volume, attenuation in Hounsfield units presented as an interval for patients with inhomogeneous hematomas, and midline shift.

**FIG. 4.**
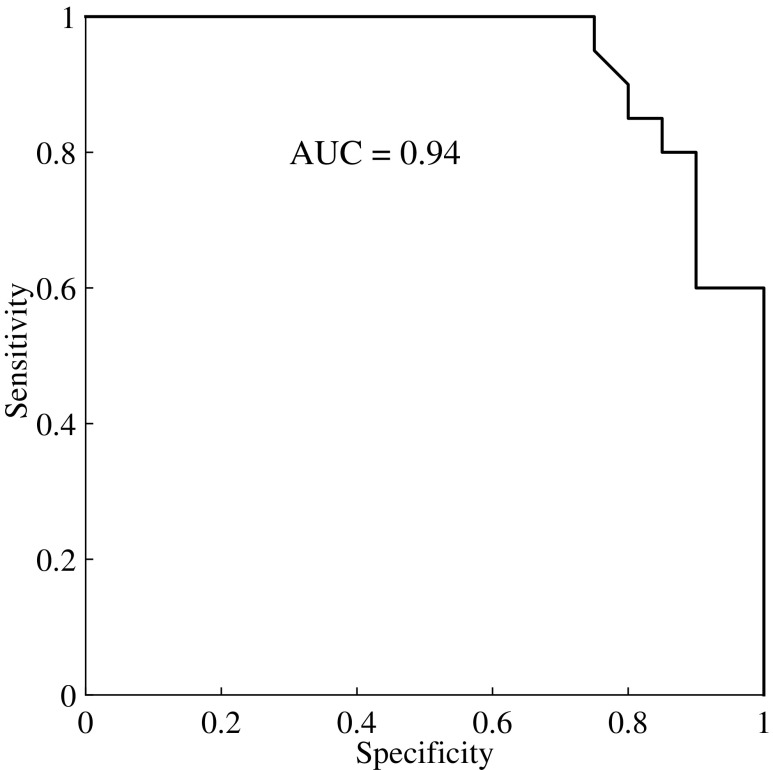
The receiver operating characteristic curve and area under the curve (AUC) value for the leave-one-out cross-validation procedure.

**FIG. 5.**
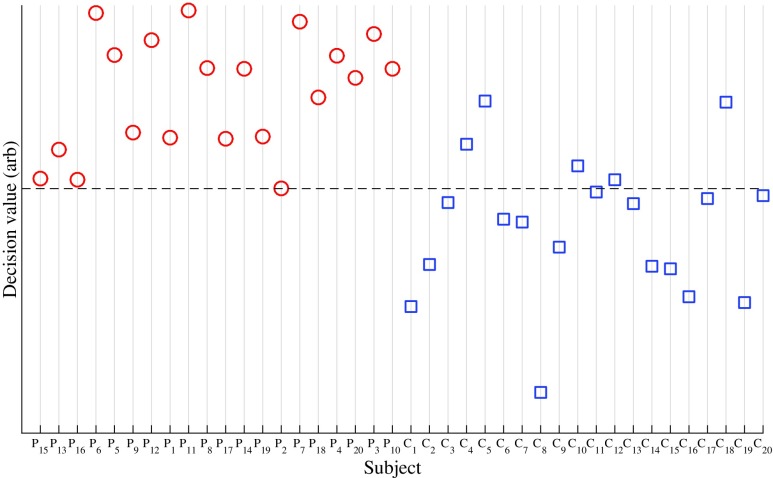
Scatter plot of the classifier decision value for all subjects. The patients have been plotted in increasing order of total hematoma size (for patients with bilateral hematoma, the left and right side volumes were summarized) from left to right. The controls are plotted in chronological order from left to right. The dashed line shows the decision value for the classifier yielding 100% sensitivity at 75% specificity. The receiver operating characteristic curve (Fig. 4) was constructed by adjusting the decision value—i.e., moving the dashed line and calculating the sensitivity and specificity for all possible decision values.

Figure 4 shows the ROC curve for differentiating cSDH from HC. The AUC is 0.94, with a specificity of 75% at 100% sensitivity. Figure 5 shows the scatter plot.

Table 3 shows the results of the tests for robustness and bias of the classification.

**Table 3.** Results for Test of Robustness and Bias of the Classification

*Data sets*	*Mean AUC ± SD*
Remove two random subjects (*n* = 100)	0.90 ± 0.03
Random diagnosis (*n* = 100)	0.47 ± 0.11

AUC, area under the curve; SD, standard deviation.

## Discussion

This is the first clinical study testing the validity of MWT in identifying a traumatic intracranial lesion, cSDH. At 100% sensitivity, the specificity was 75%, which implies that the technique could be valuable for clinical triage of patients with suspected TBI.

The tests of robustness and bias of the classification indicate that the classifier was not overfitted to the data—i.e., the classification performance was good (AUC = 0.90 ± 0.03) for data sets (*n* = 100) with true diagnosis for the subjects, and classification was equal to chance (AUC ∼0.5) for data sets (*n* = 100) where subjects were randomly assigned to either the cSDH or HC group. This indicates that the MWT method with the classifier derived in this study would achieve similar performance on unseen data—i.e. on new subjects from the same populations.

### Pre-hospital screening of patients with possible TBI

There is an obvious need for a pre-hospital screening tool to identify patients with traumatic intracranial hematomas. Undertriage of patients with major trauma in general, and for severe TBI in particular, is a substantial problem.^2,4,18^ This leads to patients with major trauma receiving definitive care at non-trauma centers or delayed care at a trauma center because of need of transfer, both associated with increased mortality.^3,19^ Studies show that it is common that a large proportion of patients with major trauma are first transported to non-trauma centers.^3,18,20^

Improving triage protocols to obtain higher triage accuracy for patients with suspected TBI is a major challenge. For example, Dehli and colleagues^18^ found that the triage accuracy for major trauma (ISS >15) was almost the same, at an undertriage rate of 28%, also after a revision of the trauma team activation criteria, and that undertriaged patients had a higher frequency of neurosurgical injuries (*p* < 0.05). Another example is the study by Ashkenazi and associates^21^ that evaluated whether Glasgow Coma Scale (GCS) score or the Simplified Motor Score could identify patients with severe TBI in need of neurosurgical intervention. The results were discouraging, because there was no statistically significant difference between the proportion of patients in need of neurosurgical procedures presenting with GCS scores of 3 to 8 and GCS scores of 9 to 14 (30% vs. 27%; *p* = 0.83).^21^

Near-infrared spectroscopy, as employed by the handheld Infrascanner device, measures optical density in brain regions and has been shown to be capable of identifying intracranial hematomas.^6,22,23^ A sensitivity of 88% and specificity of 91% was demonstrated in a bi-institutional study for hematomas larger than 3.5 mL in volume and less than 2.5 cm from the surface of the brain (*n* = 365 patients, whereof 50 with hematoma), which was reduced to 69% sensitivity when all hematomas were considered (96/365 patients).^22^ The disadvantages with the method are that near-infrared light has a short tissue penetration depth, that the current configuration of distance between light source and detector limits detection to a depth of 2–3 cm, and that the device is unable to distinguish between intracranial and extracranial lesions such as periosteal hematomas.^6,23^

Another technology suggested for detection of intracranial hemorrhage is electroencephalography (EEG). Prichep and coworkers^8^ showed in a study of 394 patients with mild TBI, of which 278 were CT-negative for hemorrhage and used as control subjects and 116 had hematomas whereof 46 were included in the study (hematoma >1 mL, mean volume 16.4 mL), that a TBI-Index for EEG had a sensitivity for hematoma detection of 96% at a specificity of 44%.^8^ The performance was not influenced by the distance from the hematoma to the recording electrodes, nor by type or volume of hematoma.

The TBI-Index was derived in previous studies by employing a binary classification algorithm based on selected quantitative features from an EEG limited to five electrodes placed on the forehead. Compared with the Infrascanner, the sensitivity for EEG appears to be higher for small bleedings, and a larger penetration depth appears to be an advantage with EEG. The specificity of EEG is poor, however.

The microwave technique used in this study allows for a portable device to be used in a pre-hospital setting and to identify an intracranial hematoma at the scene of the injury, thereby enhancing early triage and allowing for more adequate early care. In the current pilot study, we found a specificity of 75% at a sensitivity of 100% for differentiating cSDH from HC. Transferring these results to a pre-hospital setting means that all patients with a significant intracranial hemorrhage would be detected. The rate of undertriage and the time to possible neurosurgical intervention for patients with TBI could thus be reduced.

The cost for detecting all patients with hemorrhages would be 25% false positives—i.e., some patients would be overtriaged. When compared with current clinical practice, however, exemplified by the results from Lecky and associates,^4^ presenting an “overtriage ratio of 13:1 for neurosurgery and 4:1 for TBI,” these results are not discouraging. Moreover, MWT has the potential to be used in hospitals to reduce the amount of unnecessary CT scans for patients with mild TBI; to monitor patients in the neurosurgical intensive care unit—e.g., to follow the progression of hematomas.^7^

In the future, it may also be possible to image the brain and visualize hemorrhage in real time.^24^ Currently, MWT images do not provide detailed anatomical information compared with CT, and the time required to derive an image is too long for field use without employing *a priori* information about the patient, such as a previous magnetic resonance (MR) image.^25^ Imaging methods for MWT typically require hours to produce an image, although development of faster algorithms is ongoing.^26,27^ In contrast, the classification approach used in the present study is fast, giving a result within seconds. The main advantages with using a diagnostic algorithm is that results are presented in real time, and that no operator image interpretation is necessary.

### Limitations of the study

Because of limitations in the current study, further trials are needed before the value of MWT for pre-hospital triage of TBI can be assessed fully. Here, we have included only patients with cSDH, a diagnosis seldom seen in the pre-hospital emergency setting. Because this was the first study of patients with traumatic intracranial hemorrhage, we focused on patients who did not need immediate neurosurgical intervention. Obviously, for the acute trauma setting, it would be more interesting to include patients with acute hematomas. Further studies are under way to test the device in patients with acute hematomas (e.g., ClinicalTrials.gov, identifier: NCT02728908). Further, to work properly in a clinical setting, the device must also be tested for patients with other intracranial pathologies (e.g., hydrocephalus) or foreign objects implanted (e.g.. shunts or aneurysm clips), which were excluded in this investigation.

The cSDH in the current study were all large (mean total hematoma volume [left and right side summed if bilateral] = 112 mL), because of the study design of recruiting patients admitted for surgery of cSDH, and usually acute intracranial hemorrhages are smaller. This raises the question whether the device would miss smaller hematomas. The cSDH in the current study showed a large variability in attenuation (Table 2), because of different amounts of acute blood, blood breakdown products, and membranes. This means that the dielectric properties of cSDH vary. Although the cSDH in this study are large, their heterogeneous composition may make them more difficult to detect than acute hematomas. Future studies are needed to assess the dielectric properties of different types of brain hemorrhage.

Further, the concept study by Candefjord and colleagues,^7^ using tests on a phantom of subdural hematoma and numerical simulations, shows that the classifier requires a training data set size in the order of 100 patients and 100 control subjects to achieve high accuracy, because of high patient intervariability regarding factors such as head size.^7^ This indicates that a larger clinical study would allow for developing a diagnostic algorithm with capacity to detect all clinically significant hematomas, without causing a large number of false positives. The first clinical study showing that MWT can differentiate hemorrhagic and ischemic stroke, where hematoma volumes ranged from under 1 mL to 280 mL, further supports this notion.^15^

Numerical simulations have shown that MWT can be valuable for estimating the size and position of the hematoma.^7,28^ In the current study, no clear relation between decision value and hematoma volume was found (cf. Fig. 3), but such a relationship would be expected in a large clinical study of acute traumatic intracranial hemorrhage.

Those in the HC group were classified as “healthy” because they had no significant medical history and showed no signs of neurologic disorder. The HC subjects, however, could still have intracranial pathology (including cSDH) because no imaging was available for comparison. The nature of the investigation did not allow for blinding of the patient or the examiner.

The diagnostic algorithm has not been evaluated in an independent patient cohort in this pilot study. Although tests of robustness and bias have been performed and indicate that the results would be applicable to a new patient cohort, future prospective studies are needed to confirm the validity of MWT in conjunction with a diagnostic algorithm on unseen patients.

## Conclusion

This is the first clinical study testing the validity of MWT in detecting a traumatic intracranial lesion (cSDH). At 100% sensitivity, the specificity was 75%. The device is portable and takes measurements in 45 sec, which allows for pre-hospital use. The results show promise for the device to enhance triage, aid in decision making in the emergency department, and to provide additional information in monitoring of patients in the intensive care unit. Further investigations are under way to test the capacity of the device to identify different other intracranial lesions.
